# Seasonal Dynamics and Spatial Distribution of *Aedes albopictus* (Diptera: Culicidae) in a Temperate Region in Europe, Southern Portugal

**DOI:** 10.3390/ijerph17197083

**Published:** 2020-09-27

**Authors:** Hugo C. Osório, Jorge Rocha, Rita Roquette, Nélia M. Guerreiro, Líbia Zé-Zé, Fátima Amaro, Manuel Silva, Maria João Alves

**Affiliations:** 1 Centre for Vectors and Infectious Diseases Research/National Institute of Health Doutor Ricardo Jorge, Avenida da Liberdade 5, 2965-575 Águas de Moura, Portugal; libia.zeze@insa.min-saude.pt (L.Z.-Z.); fatima.amaro@insa.min-saude.pt (F.A.); manuel.silva@insa.min-saude.pt (M.S.); m.joao.alves@insa.min-saude.pt (M.J.A.); 2 Instituto de Saúde Ambiental, Faculty of Medicine, University of Lisbon, Av. Prof. Egas Moniz, Ed. Egas Moniz, Piso0, Ala C, 1649-028 Lisboa, Portugal; 3 Institute of Geography and Spatial Planning, University of Lisbon, Rua Branca Edmée Marques, 1600-276 Lisboa, Portugal; jorge.rocha@campus.ul.pt; 4 Department of Epidemiology/National Institute of Health Doutor Ricardo Jorge, Avenida Padre Cruz, 1649-019 Lisboa, Portugal; rita.roquette@insa.min-saude.pt; 5 NOVA Information Management School, Campus de Campolide, 1070-312 Lisboa, Portugal; 6 Department of Public Health and Planning, Algarve Regional Health Administration, IP, Rua Brites de Almeida, n° 6, 3rd Dt° 8000-234 Faro, Portugal; nguerreiro@arsalgarve.min-saude.pt; 7 BioISI—Biosystems & Integrative Sciences Institute, Faculty of Sciences, University of Lisbon, 1749-016 Lisbon, Portugal

**Keywords:** *Aedes albopictus*, invasive mosquitoes, population dynamics, arboviruses, Portugal

## Abstract

*Aedes albopictus* is an invasive mosquito that has colonized several European countries as well as Portugal, where it was detected for the first time in 2017. To increase the knowledge of *Ae. albopictus* population dynamics, a survey was carried out in the municipality of Loulé, Algarve, a Southern temperate region of Portugal, throughout 2019, with Biogents Sentinel traps (BGS traps) and ovitraps. More than 19,000 eggs and 400 adults were identified from May 9 (week 19) and December 16 (week 50). A positive correlation between the number of females captured in the BGS traps and the number of eggs collected in ovitraps was found. The start of activity of *A. albopictus* in May corresponded to an average minimum temperature above 13.0 °C and an average maximum temperature of 26.2 °C. The abundance peak of this *A. albopictus* population was identified from September to November. The positive effect of temperature on the seasonal activity of the adult population observed highlight the importance of climate change in affecting the occurrence, abundance, and distribution patterns of this species. The continuously monitoring activities currently ongoing point to an established population of *A. albopictus* in Loulé, Algarve, in a dispersion process to other regions of Portugal and raises concern for future outbreaks of mosquito-borne diseases associated with this invasive mosquito species.

## 1. Introduction

*Aedes albopictus* (Skuse, 1894) is an invasive mosquito species of major concern to public health because of its vector role in the transmission of several arboviruses, such as chikungunya, dengue, and Zika [1]. In recent decades, *A. albopictus* has spread throughout the world and is now found on all continents except Antarctica [2]. This worldwide successful expansion has been promoted mainly by human trade via passive transport of eggs on used tires and ornamental plants such as “lucky bamboo” together with passive transportation of adult mosquitoes by public and private ground transport from heavily infested areas [3,4]. In Europe, this species was first detected in Albania in 1979 [5], and, since its finding in Italy in 1990, *A. albopictus* has been found to be spreading, having already been introduced in 25 European countries and established in 19 of them [6,7]. Portugal has always been on the list of the most likely regions for introducing and establishing *A. albopictus* [2], and, as expected, in 2017, two independent introduction events were reported for the first time [8,9].

Although autochthonous transmission of dengue and chikungunya has not yet been reported in mainland Portugal, autochthonous transmission in Europe related to *A. albopictus* is known since 2007, when an outbreak of chikungunya occurred in the region of Emilia Romagna in Italy [10,11]. More recently, chikungunya outbreaks have been reported in France in 2010 [12,13], 2014 [14], and 2017 [15], and, again, in Italy in 2017 [16]. Autochthonous dengue cases caused by dengue serotypes 1 and 2 have also been reported in 2010 in Croatia and France [17,18], and in France in 2013 [19], 2014 [20], and 2015 [21]. In 2018, 12 cases of autochthonous dengue were confirmed in the European Union (EU), six in Spain (five in the region of Murcia and one in Catalonia), and six in France (five cases in Saint Laurent du Var with one case in Montpellier) [22].

To mitigate the potential impact of *A. albopictus* in transmitting human diseases, efforts must be made to better understand the biology and the ecology of the species. The spatial and temporal characterization of *A. albopictus* wild populations based on baseline data is required prior to the initiation of any control strategy in an Area-Wide-Integrated Pest Management (AW-IPM) program. For this purpose, after identifying the target distribution area, baseline data should be collected longitudinally to characterize the phenology and distribution of target vector populations [23,24,25]. The data collection is used to formulate adjusted vector control strategies on the target areas by reducing vector abundance and preventing outbreaks of mosquito-borne diseases related to *A. albopictus*.

Temperature is known to be a crucial driver for *A. albopictus* activity at different levels, from adult abundance and biting behavior to oviposition activity [26,27]. Previous studies showed that the seasonal emergence of host-seeking females was strongly influenced by the minimum temperature, and a lower threshold of 13 °C was identified [27]. The prediction of starting the activity in the seasonal dynamics of *A. albopictus* promote timely operational interventions that will prevent mosquito densities from rising above the risk threshold for arboviruses outbreaks in the areas colonized by *A. albopictus*. Therefore, it is of the utmost importance to integrate the monitoring of environmental factors as temperature in vector surveillance.

In Portugal, a National Vector Surveillance Network—REVIVE (REde de VIgilância de VEctores)—established in 2008 under the custody of the Portuguese Ministry of Health is responsible for the mosquito monitoring and arboviral screening in field-collected arthropod vectors [28]. At airports, ports, storage areas, and specific border regions with Spain and locations where *A. aegypti* or *A. albopictus* are present, monitoring takes place throughout the year with the commitment of local and regional authorities. Under the frame of REVIVE, the *A. albopictus* activity in the Algarve region has been monitored since 2017. This study aimed to characterize the seasonal activity of this recently introduced species and to address the following questions: (1) correlation of egg and adult sampling, (2) species seasonal abundance peak in the target region, and (3) correlation between temperature and mosquito abundance and identification of temperature thresholds for *A. albopictus* occurrence. The results and discussion in this study represent the initial effort to provide the required baseline information in strategic control interventions and environmental policies to effectively manage mosquitoes and, consequently, prevent vector-borne diseases.

## 2. Materials and Methods

### 2.1. Study Area and Mosquito Collection

**Figure 1:**
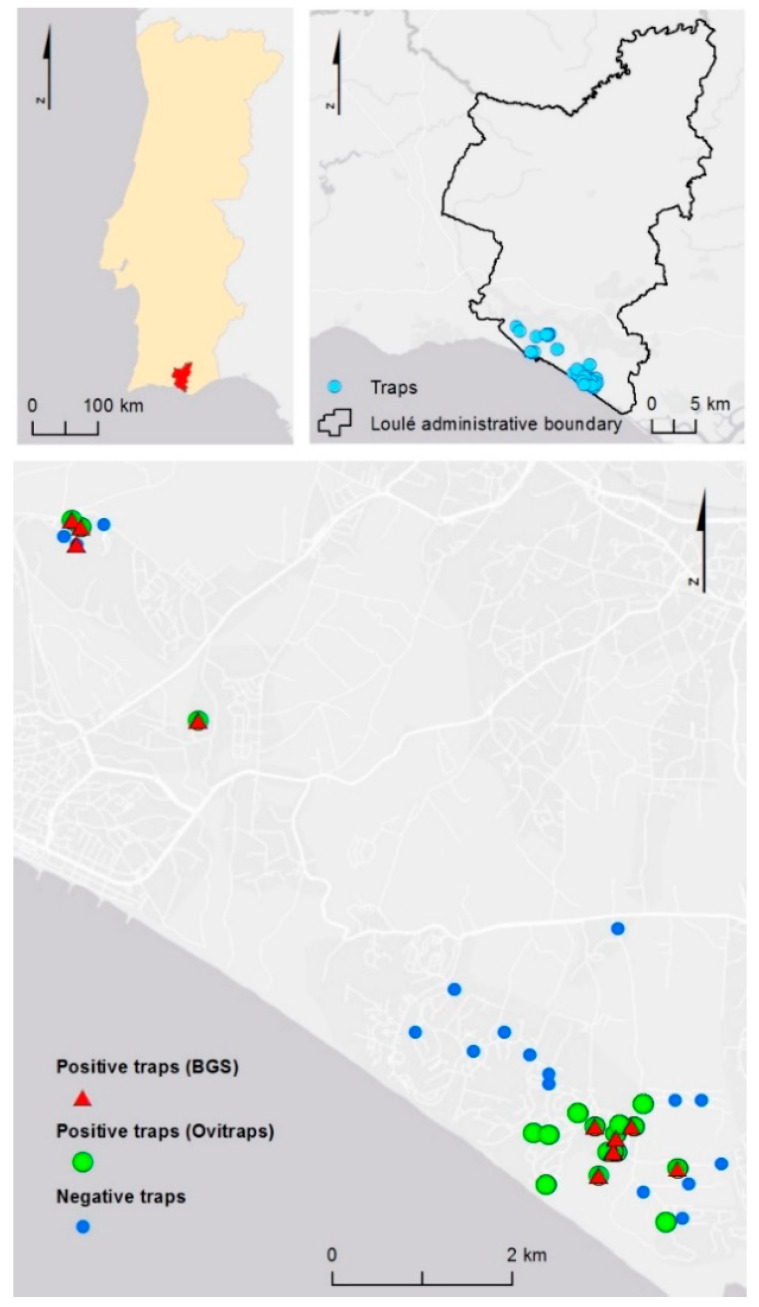
*Aedes albopictus* monitoring stations in the municipality of Loulé, Algarve, Portugal. Eight BG-sentinel traps baited with BG-lure (BGS, Biogents, Germany) and 36 Ovitraps were used during the study period. Positive traps for *Aedes albopictus*: red triangles = BGS traps, green dots = Ovitraps.

Monitoring activities were carried in the hotspot for *A. albopictus* in the municipality of Loulé (LAO 1), parishes of Quarteira and Almancil (LAU 2) in Algarve region, from 24 May 2017, to 7 February 2020 (Figure 1). During 2019, adult mosquitoes’ activity was monitored using eight BG-sentinel traps (Biogents Sentinel traps—BGS traps) baited with BG-lure (Biogents, Regensburg, Germany). Oviposition activity was monitored by using 30–36 ovitraps that consisted of small black plastic buckets with 1–L of water with no attractant and provided with an oviposition support, which is usually a germination paper on a plastic stick. All traps in permanent monitoring stations were weekly inspected for the presence of adult mosquitoes and eggs year-round (Table 1; Table S1). In positive ovitraps, the oviposition paper was placed in collection tubes and sent to lab for species confirmation and counting. A new oviposition paper and fresh water were replaced after cleaning by scrubbing the inner wall of the bucket. Adult mosquitoes captured in the BGS traps were aspirated, transferred alive to collection tubes, and sent to the laboratory. All mosquito samples were collected by the national REVIVE surveillance network at public and private proprieties with the respective accountable knowledge and permission. The mosquitoes were identified using the identification keys of Ribeiro and Ramos [29] and Schaffner et al. [30].

Minimum and maximum values of temperature were collected in situ during the monitoring activities with field thermometers at the permanent monitoring stations on a weekly basis.

### 2.2. Arboviruses Screening

Considering their importance as vectors for several important arboviruses, adult mosquitoes were screened by real-time polymerase chain reaction (PCR) for chikungunya, dengue, and Zika viruses using the RealStar^®^ RT-PCR kits (altona Diagnostics GmbH, Hamburg, Germany), and by Pan-flavi NS5 conventional RT-PCR to include other potential flaviviruses [31,32]. Conventional RT-PCR amplicons were observed on 1.5% agarose gels.

### 2.3. Data Analysis

The maps and the *A. albopictus* population density analysis based on egg number and adult’s collection numbers were performed with ArcGIS 10.8 (ESRI, Redlands, CA, USA.).

Statistical analysis was performed using R version 3.6.3. (R Core Team, www.r-project.org), and, in particular, the tseries package [33]. Sample cross correlation function (CCF) was used to detect lag in the independent variable (x_t_) that works as a predictor of the dependent variable (yt). CCF works upon a set of sample correlations among x_t+h_ and y_t_, with h = 0, ±1, ±2, …, ±n. If h is negative, it shows a correlation between the value of the dependent variable at a specific moment (t) and the ones of the independent variable some time lags before (t − h). All significant differences and positive correlations were based on the *p*-value (p). They were considered significant when *p* < 0.05.

Spatial analysis was performed through a kernel density estimation (KDE) density analysis and the following occurrence estimation. KDE is a non-parametric method for modelling the probability density function through inferences on data based on a finite sample. While the average flight range of *A. albopictus* is estimated to be 200 m, in some environments, it has the ability to disperse over a wider area between breeding site and hosts [34,35]. A 1000-m neighborhood distance was considered in our analysis.

## 3. Results

Ten mosquito species were identified during the study either in the adult or in the larvae stage: *Anopheles maculipennis* s.l. (*N* = 9 females), *Culiseta annulata* (*N* = 10 larvae), *C. longiareolata* (*N* = 616 larvae, *N* = 8 females, *N* = 22 males), *Culex modestus* (*N* = 1 female), *C. univittatus* (*N* = 7 females, *N* =1 male), *C. pipiens* (*N* = 243 larvae, *N* = 286 females), *C. theileri* (*N* = 20 females), *Ochlerotatus caspius* (*N* = 20 femles), and *O. detritus* (*N* = 10 females). *Aedes albopictus* was identified by the REVIVE for the first time in 2018 in the municipality of Loulé (LAU 1), initially in Vilamoura (Quarteira, LAU 2), and later in Vale do Garrão, Vale do Lobo, and Quinta do Lago (Almancil, LAU 2) (Figure 1).

**Figure 2:**
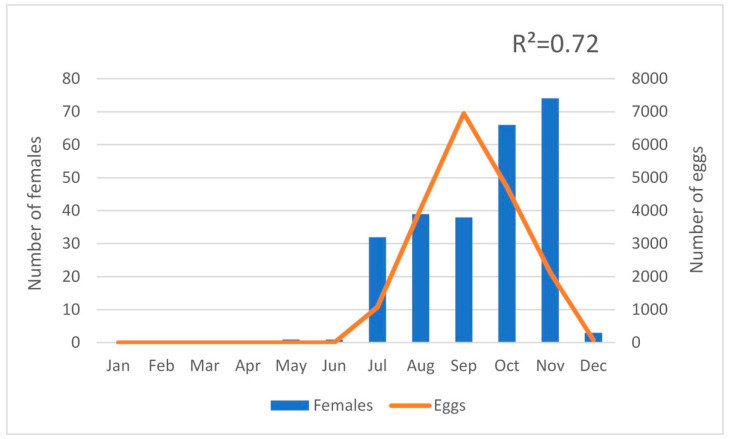
Positive correlation between the number of *Aedes albopictus* females captured in the BGS traps and eggs counted in the ovitraps during 2019.

Ninety-seven adults, 67 females, and 30 males were collected in BGS traps and human landing collections from 12th July to 5th November. During this year, the trap network was adjusted and set according to the distribution data obtained. In 2019, 19,004 eggs and 449 adults were identified with 330 females and 119 males (Table 1). The first sign of *A. albopictus* activity was on May 9 (week 19) and the last sign was on December 16 (week 50) when the last specimens of 2019 were collected in the BGS traps. The abundance peak of *A. albopictus* population was identified in the months from September to November, which corresponds September to the high number of eggs counted in ovitraps (week 36–39, *N* = 6949) and November to the high number of adult mosquitoes collected in the BGS traps (week 45–48, *N* = 74 females). Actually, a strong positive correlation (R^2^ = 0.72) was found between the number of eggs and the number of females collected (Figure 2).

**Figure 3:**
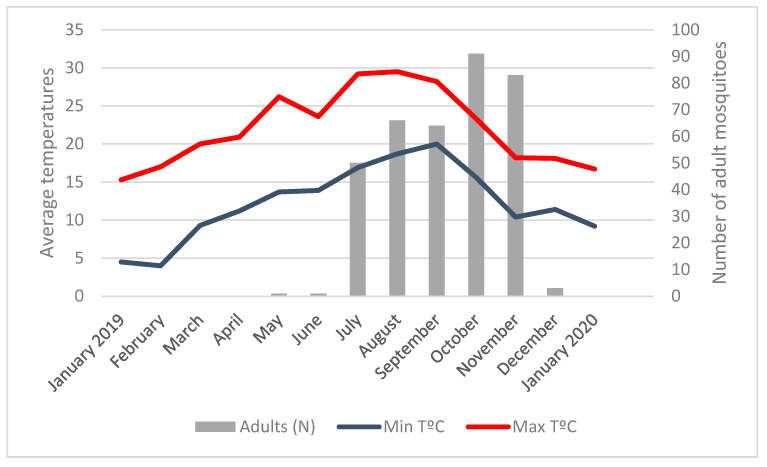
Number of adult *Aedes albopictus* mosquitoes and average minimum and maximum temperatures.

The first collections of *A. albopictus* in May corresponded to an average minimum temperature of 13.7 °C and an average maximum temperature of 26.2 °C (Figure 3; Table S2). October recorded a 15.6 °C average minimum temperature and November recorded a 10.4 °C average minimum temperature. From mid-December, when the average minimum temperature was 10.3 °C. *A. albopictus* activity ceased and no more adult mosquitoes or eggs were found until April 2020, when the average minimum temperature recorded was again above 13 °C (13.6 °C).

**Figure 4:**
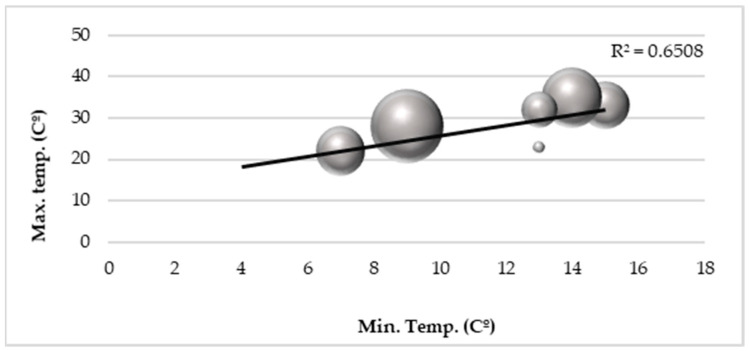
Correlation between the number of adult mosquitoes (circles) and minimum and maximum temperatures.

A positive correlation between temperature and *A. albopictus* abundance was also found with higher temperatures favoring mosquito abundance. Minimum temperatures did not have a direct influence on the number of mosquitoes, but worked indirectly through the thermal amplitude when mosquito occurrences in different maximum and minimum temperature conditions were observed (R^2^ = 0.65) (Figure 4).

**Figure 5:**
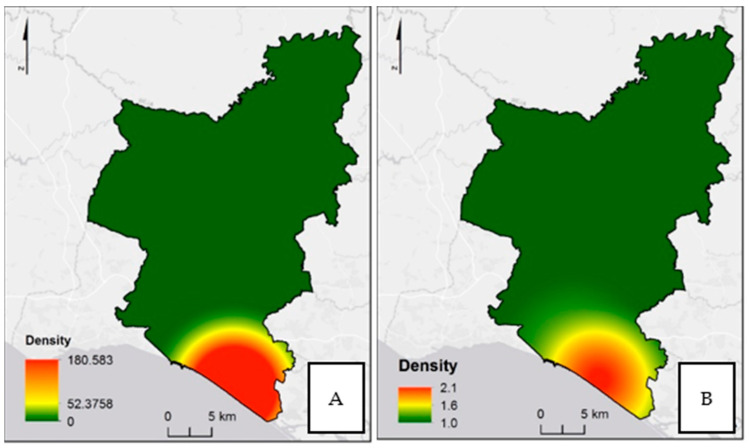
Density of *Aedes albopictus* population: (**A**) analyses based on the egg number, and (**B**) analyses based on the adult number. All densities are in number by square kilometer.

In the analysis of the *A. albopictus* population density geographic distribution, the highest abundances were observed in the southeast, Almancil (LAU 2), either in the analyses based on egg or adults’ numbers (Figure 5). However, a wider area of distribution was found on the analysis based on the number of eggs (Figure 5A).

Regarding arboviruses screening, 172 female mosquitoes were analyzed. Chikungunya virus and pathogenic flaviviruses were not detected.

## 4. Discussion

The present study describes the field investigation aimed at analyzing the *A. albopictus* seasonality and distribution in a temperate region in Southern Portugal, Algarve, where this species was reported for the first time in 2017 [9]. Since then, the target region was always under entomological monitoring and a better understanding of the geographic distribution allowed the setup of permanent vector surveillance stations to monitor seasonal activity using BGS traps for adults and ovitraps [8]. The strong correlation of *A. albopictus* female’s abundance and the egg index identified in this study represents a valuable tool in mosquito monitoring and surveillance of vector-borne diseases associated with abundance of the vector population assessed. Based on ovitrap’s egg frequency, the abundance of adult females can be predicted as the *A. albopictus* host-seeker activity since it has already been demonstrated that these two variables are related [27]. The use of the egg index in vector control activities may contribute just by itself for timely interventions to suppress the adult population, as already indicated in previous studies, while corroborating the importance of using ovitraps in vector control operations [36,37,38,39].

The seasonal dynamic of *A. albopictus* in Algarve was similar to those reported in other regions of southern Europe, where the abundance peaks are recorded at the end of the hot season [40]. It was observed in this study that the seasonal emergence of host-seeking females was when the average minimum temperature was above 13 °C, which was recorded for the first time in May 2019, triggering the activity of the *A. albopictus* in the region. This observation confirms the temperature threshold of 13.0 °C identified for European female *A. albopictus* populations in the field necessary to initiate activity in adult female mosquitoes [27,41]. In December 2019, the activity of *A. albopictus* in Algarve ceased, and no more eggs or adult mosquitoes were found until April 2020, when the average minimum temperature registered in the region was above 13.0 °C. A temperature threshold identified for the end of adult activity was 9.0 °C. However, the Algarve population of *A. albopictus* ceased its activity when the average minimum temperature in the region was 10.3 °C. Nonetheless, mosquitoes can be affected by microclimate conditions and other environmental factors that were not addressed in this study, namely precipitation and humidity [42].

Particularly on the *A. albopictus* female activity, a positive effect of temperature was identified, which corroborates the results obtained in previous studies [27,43,44]. The abundance peak was identified from September to November, corresponding September to the highest number of eggs collected and November to the highest abundance of the adult population, as it would be expected.

The results in this study also show a pattern of population density that can represent the invasion process, namely the mechanism of active mosquito dispersion. A higher density of eggs in a broader area relatively to the adult population density may reflect layers of active dispersion in the field. These data points to an established *A. albopictus* population in a process of active/passive dispersion in the south of Portugal, which is a region of Algarve.

The spread of *A. albopictus* throughout Europe has raised concern for the future outbreaks of mosquito-borne disease. Arbovirus outbreaks typically occur 5–15 years after *A. aegypti* or *A. albopictus* detection [2,45]. The presence of *A. albopictus* in Portugal and its establishment is a major public health threat, raising concern about the introduction of several arboviruses.

Monitoring at regional scale to access baseline data on distribution areas and seasonality for this invasive species is a valuable tool in elaborating the strategic control measures that may contribute to the vector population suppression in an integrated plan and prevents mosquito borne diseases. The results in this study provide useful indicators to prioritize public mosquito control measures in temperate urban areas where nuisance, human-mosquito contact, and risk of local arbovirus transmission are likely higher. Other environmental factors than temperature can affect mosquito phenology. For instance, relative humidity and precipitation play a role in *A. albopictus* seasonality and distribution. Future studies should include these variables in the analysis. Further experimental and modeling work is now needed to predict how this species will change its spatial distribution and seasonal activity in relation to changes in climate and land use in Portugal.

## 5. Conclusions

The results of this study suggest that an *A. albopictus* population is established and dispersed in the region of Algarve. The activity of this species started when the average of minimum temperatures was above 13 °C, which corroborates previous studies on *A. albopictus* seasonality in Europe. The abundance peak was identified in the September to November period. According to our results and the local environmental conditions, vector control efforts should consider the mosquito presence between May and December and start in April.

Egg and adult abundance of Algarve’s *A. albopictus* were positively correlated, which represents an important tool in mosquito monitoring and surveillance. A positive effect of temperature on seasonal activity of the adult population (female activity) was also observed, as shown how climate change may affect the abundance and distribution patterns of this population. Its presence in the region since 2017, the results in this study, and the continuously monitoring activities currently ongoing point to an established population of *A. albopictus* in Loulé, Algarve, and on dispersion to other regions of Portugal.

## Figures and Tables

**Table 1:** Number of *Aedes albopictus* eggs, females, and males collected from week one to week 53 (January–December) 2019.

Week	Eggs (*N*)	Eggs/Ovitrap	Females	Females/BGS * Trap	Males	Males/BGS Trap
1–18		0		0		0
19		0	1	0.1		0
20		0		0		0
21		0		0		0
22		0		0		0
23		0	2	0.3		0
24		0		0		0
25		0	3	0.4		0
26		0		0		0
27		0		0		0
28		0	16	2	1	0.1
29	526	14.6	4	0.5	5	0.6
30		0	13	1.6	9	1.1
31	1015	28.2	40	5	3	0.4
32	840	23.3	15	1.9	10	1.3
33	625	17.4	3	0.4	5	0.6
34	1393	38.7	6	0.8	6	0.8
35	732	20.3	14	1.8	6	0.8
36	2143	59.5	30	3.8	16	2
37	2375	66	16	2	8	1
38	739	20.5	1	0.1		0
39	762	21.2	16	2	13	1.6
40	1472	40.9	14	1.8	20	2.5
41	1812	50.3	35	4.4	1	0.1
42	1421	39.5	7	0.9		0
43	561	15.6	9	1.1	6	0.8
44	372	10.3	8	1	1	0.1
45	1338	37.2	41	5.1	7	0.9
46	517	14.4	26	3.3	2	0.3
47	20	0.6		0		0
48	256	7.1	7	0.9		0
49	85	2.4	1	0.1		0
50		0	2	0.3		0
51–53		0		0		0
**Total**	**19,004**	**528**	**330**	**41**	**119**	**15**

* Biogents Sentinel.

## Supplementary Materials

The following are available online at https://www.mdpi.com/1660-4601/17/19/7083/s1. Table S1: Collection sites and GPS locations for ovitraps and BG-sentinel traps. Table S2: Local average minimum and maximum temperatures and number of mosquitoes per month.
