# Supplementary material for: Seasonal Dynamics and Spatial Distribution of Aedes albopictus (Diptera: Culicidae) in a Temperate Region in Europe, Southern Portugal
Source: Int J Environ Res Public Health. 2020 Sep 27;17(19):7083. doi: 10.3390/ijerph17197083 (PMC7579007; doi:10.3390/ijerph17197083)
Supplement: Supplementary file 1 [file ijerph-17-07083-s001.zip › ijerph-935258-supplementary.pdf]

Table S1. Collection sites and GPS locations for ovitraps and BG-sentinel traps.

**Program**	**Trap**	**Code**	**X**	**Y**
PRALGARVE_BGS	BGS	PALVIL_BGS	37º05'36.7"N	8º05'49.2"W
PRALGARVE_BGS	BGS	QTRBGS_17	37°04'39.1"N	8°05'03.9"W
PRALGARVE_BGS	BGS	VGBGS_1	37º02'35.9"N	8º02'34.8"W
PRALGARVE_BGS	BGS	VGBGS_4	37º02'39.7"N	8º02'34.0"W
PRALGARVE_BGS	BGS	VGBGS_7	37º02'42.9"N	8º02'41.3"W
PRALGARVE_BGS	BGS	VGBGS_8	37°02'43.0"N	8°02'28.4"W
PRALGARVE_BGS	BGS	VSP_BGS	37º05'29.7"N	08º05'47.4"W
PRALGARVE_BGS	BGS	VSP_BGS2	37º05'34.6"N	8º05'45.9"W
PRALGARVE_OV	Ovitraps	PALVIL_OV	37º05'36.7"N	8º05'49.2"W
PRALGARVE_OV	Ovitraps	PALVIL_OV6	37º05'32.1"N	8º05'52.1"W
PRALGARVE_OV	Ovitraps	QTROV_17	37°04'39.1"N	8°05'03.9"W
PRALGARVE_OV	Ovitraps	QTROV_29	37°04'54.9"N	8°05'01.5"W
PRALGARVE_OV	Ovitraps	QTROV_30	37°04'36.3"N	8°05'16.4"W
PRALGARVE_OV	Ovitraps	QTROV_31	37°04'48.0"N	8°05'14.4"W
PRALGARVE_OV	Ovitraps	QTROV_32	37°04'23.4"N	8°05'00.7"W
PRALGARVE_OV	Ovitraps	QTROV_33	37°04'16.7"N	8°04'58.8"W
PRALGARVE_OV	Ovitraps	VGOV_1	37º02'35.9"N	8º02'34.8"W
PRALGARVE_OV	Ovitraps	VGOV_10	37°02'49.5"N	8°02'24.2"W
PRALGARVE_OV	Ovitraps	VGOV_11	37°02'40.7"N	8°02'58.1"W
PRALGARVE_OV	Ovitraps	VGOV_12	37°02'41.1"N	8°03'03.7"W
PRALGARVE_OV	Ovitraps	VGOV_13	37°02'26.4"N	8°02'59.1"W
PRALGARVE_OV	Ovitraps	VGOV_14	37°02'15.7"N	8°02'16.0"W
PRALGARVE_OV	Ovitraps	VGOV_15	37°02'31.1"N	8°02'11.7"W
PRALGARVE_OV	Ovitraps	VGOV_16	37°02'35.8"N	8°02'37.0"W
PRALGARVE_OV	Ovitraps	VGOV_18	37°03'39.6"N	8°02'33.4"W
PRALGARVE_OV	Ovitraps	VGOV_19	37°02'50.4"N	8°02'12.9"W
PRALGARVE_OV	Ovitraps	VGOV_2	37º02'35.8"N	8º02'35.0"W
PRALGARVE_OV	Ovitraps	VGOV_20	37°02'48.6"N	8°02'04.6"W
PRALGARVE_OV	Ovitraps	VGOV_21	37°02'31.0"N	8°01'56.2"W

PRALGARVE_OV	Ovitraps	VGOV_22	37°02'16.8"N	8°02'10.1"W
PRALGARVE_OV	Ovitraps	VGOV_23	37°02'26.4"N	8°02'11.2"W
PRALGARVE_OV	Ovitraps	VGOV_24	37°02'55.2"N	8°02'58.2"W
PRALGARVE_OV	Ovitraps	VGOV_25	37°03'03.6"N	8°03'04.8"W
PRALGARVE_OV	Ovitraps	VGOV_26	37°03'10.0"N	8°03'14.0"W
PRALGARVE_OV	Ovitraps	VGOV_27	37°03'04.5"N	8°03'25.2"W
PRALGARVE_OV	Ovitraps	VGOV_28	37°03'22.2"N	8°03'32.0"W
PRALGARVE_OV	Ovitraps	VGOV_3	37º02'35.8"N	8º02'33.7"W
PRALGARVE_OV	Ovitraps	VGOV_5	37º02'43.5"N	8º02'32.8"W
PRALGARVE_OV	Ovitraps	VGOV_6	37°02'40.8"N	8°02'33.8"W
PRALGARVE_OV	Ovitraps	VGOV_7	37º02'42.9"N	8º02'41.3"W
PRALGARVE_OV	Ovitraps	VGOV_8	37°02'43.1"N	8°02'27.3"W
PRALGARVE_OV	Ovitraps	VGOV_9	37°02'46.9"N	8°02'47.5"W
PRALGARVE_OV	Ovitraps	VSP_OV	37º05'29.7"N	08º05'47.4"W
PRALGARVE_OV	Ovitraps	VSP_OV2	37º05'34.6"N	8º05'45.9"W

Table S2. Local average minimum and maximum temperatures and number of mosquitoes per month.

	**Average temperatures**						
	**Min TºC**	**Max TºC**	**Average**	**Eggs (N)**	**Females (N)**	**Males (N)**	**Adults (N)**
January 2019	4,5	15,3	9,9				0
February	4	17	10,5				0
March	9,3	20	14,65				0
April	11,2	20,9	16,05				0
May	13,7	26,2	19,95		1		1
June	13,9	23,6	18,75		1		1
July	16,9	29,2	23,05	1075	32	18	50
August	18,7	29,5	24,1	4056	39	27	66
September	20	28,2	24,1	6949	38	26	64
October	15,6	23,3	19,45	4708	66	25	91
November	10,4	18,2	14,3	2131	74	9	83
December	11,4	18,1	14,75	85	3		3

January 2020	9,2	16,7	12,95				0
